# Targeting Lysine Deacetylases (KDACs) in Parasites

**DOI:** 10.1371/journal.pntd.0004026

**Published:** 2015-09-24

**Authors:** Qi Wang, Bruce A. Rosa, Bakela Nare, Kerrie Powell, Sergio Valente, Dante Rotili, Antonello Mai, Garland R. Marshall, Makedonka Mitreva

**Affiliations:** 1 The Genome Institute, Washington University School of Medicine, St. Louis, Missouri, United States of America; 2 SCYNEXIS, Inc, Research Triangle Park, North Carolina, United States of America; 3 Istituto Pasteur-Fondazione Cenci Bolognetti, Dipartimento di Chimica e Tecnologie del Farmaco, Università degli Studi di Roma “La Sapienza”, Roma, Italy; 4 Department of Biochemistry and Molecular Biophysics, Washington University in St. Louis, St. Louis, Missouri, United States of America; 5 Departments of Genetics and of Internal Medicine, Washington University School of Medicine, St. Louis, Missouri, United States of America; McGill University, CANADA

## Abstract

Due to an increasing problem of drug resistance among almost all parasites species ranging from protists to worms, there is an urgent need to explore new drug targets and their inhibitors to provide new and effective parasitic therapeutics. In this regard, there is growing interest in exploring known drug leads of human epigenetic enzymes as potential starting points to develop novel treatments for parasitic diseases. This approach of repurposing (starting with validated targets and inhibitors) is quite attractive since it has the potential to reduce the expense of drug development and accelerate the process of developing novel drug candidates for parasite control. Lysine deacetylases (KDACs) are among the most studied epigenetic drug targets of humans, and a broad range of small-molecule inhibitors for these enzymes have been reported. In this work, we identify the KDAC protein families in representative species across important classes of parasites, screen a compound library of 23 hydroxamate- or benzamide-based small molecules KDAC inhibitors, and report their activities against a range of parasitic species, including the pathogen of malaria (*Plasmodium falciparum*), kinetoplastids (*Trypanosoma brucei* and *Leishmania donovani*), and nematodes (*Brugia malayi*, *Dirofilaria immitis* and *Haemonchus contortus*). Compound activity against parasites is compared to that observed against the mammalian cell line (L929 mouse fibroblast) in order to determine potential parasite-versus-host selectivity). The compounds showed nanomolar to sub-nanomolar potency against various parasites, and some selectivity was observed within the small panel of compounds tested. The possible binding modes of the active compounds at the different protein target sites within different species were explored by docking to homology models to help guide the discovery of more selective, parasite-specific inhibitors. This current work supports previous studies that explored the use of KDAC inhibitors in targeting *Plasmodium* to develop new anti-malarial treatments, and also pioneers experiments with these KDAC inhibitors as potential new anthelminthics. The selectivity observed begins to address the challenges of targeting specific parasitic diseases while limiting host toxicity.

## Author Summary

Due to pandemic drug resistance in the treatment of parasitic infections, there is an urgent need to identify novel drug targets and their associated drug compounds. Although “drug repurposing”, i.e. the application of known drugs and compounds to new indications such as infectious diseases, provides a cost effective approach in the development of novel therapeutics, selectivity is one of the major obstacles to overcome in getting such compounds into clinical trials as anti-parasitic drugs. Using the lysine deacetylases (KDACs) as an example, we explored the activities of a panel of known inhibitors against the KDAC targets in a range of parasitic organisms. The computational study of their binding modes to the targets (by docking the compounds to the homology models within different organisms in comparison with the human proteins) helps to rationalize the different activities observed and provide insight on the optimization of lead compounds to improve selectivity. Our work provides support of “drug repurposing” in the treatment of parasitic diseases, and demonstrates the necessity of optimizing these leads for the ultimate goal of preparing them for clinical use.

## Introduction

Neglected tropical diseases are the most common infections of the poorest populations around the globe, causing massive burdens on the countries’ general population and inhibiting economic development [1]. Treatments for these diseases usually rely on a single drug, or limited options of drugs. Most drugs used for treating neglected diseases are quite old, have unknown mechanisms of action, and often have limited effectiveness with poor safety profiles. Furthermore, drug resistance has been observed following the treatment of almost all parasitic pathogens, including protists, helminthes (roundworms) and Platyhelminthes (flatworms) [2]. Parasitic genome sequencing is now being exploited to help accelerate the development of much-needed compounds with novel mechanisms of action.

Lysine deacetylase (KDACs) is a more specific term for enzymes that remove the e-acetyl group from lysine side chains, and have emerged as an important class of drug targets with the potential to treat a variety of diseases in human, ranging from psychiatric diseases and neurodegenerative diseases to cancer [3, 4]. Given the significance of KDACs in epigenetic modulation, numerous small compounds have been developed to inhibit their activity, originally directed at altering chromatin structure and thus modulating gene expression [5]. In humans, KDACs belong to a large family with 18 members [6] divided into the Zn-dependent (Class I and Class II) and NAD-dependent (Class III) enzymes. The Zn-dependent enzymes have been the focus of intense research since they compose the majority of the KDAC family members and are the primary targets of the known inhibitors [7]. In light of “drug repurposing” for the treatment of parasitic diseases [8, 9], KDACs have been identified as an emerging drug target in all the major human parasitic pathogens [10], but no systematic characterization has been conducted to date, except in *Plasmodium* [11]. Considerable efforts were also made to utilize the collected information of the known KDAC inhibitors to explore the potential of targeting orthologs in the parasitic pathogens, ranging from *P*. *falciparum* [12–15] to *Schistosoma mansoni* [16, 17]. Andrews et al. have pursued KDAC inhibitors as antimalarial drugs [12, 18, 19]; One of their studies presents comparative gene expression profiling of *P*. *falciparum* in response to exposure to three different KDAC inhibitors [20]. Despite structural similarity between the three inhibitors, diverse transcriptional effects were observed in the study, and were attributed to possibly subtle differences in their inhibition of KDAC isoforms, or cellular distribution. Marek et al. [16] recently reported that lysine deacetylase 8 from *S*. *mansoni* (smKDAC8), the most expressed class I KDAC isoform in this organism, was a functional acetyl-L-lysine deacetylase with an essential role in parasite infectivity. Crystal structures were obtained for different inhibitors bound to smKDAC8, and their binding modes were compared for the optimization of the lead inhibitor, in order to achieve better potency and selectivity for smHDAC8 [17]. In a more recent study, many KDAC inhibitors that are currently in clinical trials for oncology were evaluated as therapeutic leads to target the kinetoplastid *Trypanosoma brucei* for human African trypanosomiasis (HAT) [21]. These inhibitors were found to be moderately to strongly potent in blocking proliferation of blood-stage *T*. *brucei* in culture; however, none of these drugs were lethal to cultured parasites when tested at human-tolerated doses. This again confirms that parasitic selectivity is the major issue to address before repurposing these drugs as anti-parasitic therapeutics [8, 10]. Similar observations were also made in other work [22]. Most of the studies to date have focused on a single enzyme isoform (or at the cellular level for the target parasite), and have neglected to explore the potential interaction of inhibitors acting on different KDAC orthologs and isoforms, in particular, the human ortholog/isoforms. In pursuing parasitic-specific or selective KDAC inhibitors, a systematic evaluation of the KDAC targets as well as their interactions with small molecular inhibitors is warranted to gain better insights at the molecular level for the improvement of inhibitor selectivity.

It should be noted that lysine deacetylases are generally referred to as histone deacetylases (HDACs; a historical imperative, as epigenetic modification of histones was described in 1964 by Allfrey et al. [23]), but it has been shown by proteomics that over 1700 proteins in cells besides histones undergo dynamic acetylation [3]. Thus, here, we use the more accurate terminology of lysine deacetylase (KDAC), acetylase (KAT) and methylase (KMT).

In this work, we took advantage of the genomes (deduced proteomes) of many parasite species (resulting from recently-emerging sequencing technologies) to characterize the KDAC family in parasites, including the less frequently-studied nematode species. KDAC family members within these species were characterized using an orthology-based approach. Most importantly, known human KDAC inhibitors were screened for activity against a few representative parasite species including the pathogen of malaria (*Plasmodium falciparum*), kinetoplastids (*T*. *brucei* and *Leishmania donovani*), and nematodes (*Brugia malayi*, *Dirofilaria immitis* and *Haemonchus contortus*). Activity observed *in vitro* against these parasites was compared with that observed for toxicity with a mammalian cell line to determine the potential for host-versus-parasite selectivity. We also examined potential molecular mechanisms by which active compounds could be acting on the targets. This work provides some perspective on the prospect of targeting KDACs in parasites and paves the way for developing more selective KDAC ligands as novel drugs to control parasitic infections of humans and animals.

## Methods

### Data Collection

Whole proteome data from 26 eukaryotic species were collected. The datasets were comprised of 11 species of nematodes, 4 species of Platyhelminthes, 5 species of protists (kinetoplastids and pathogen of malaria) and 6 species of hosts/outgroups. Data were downloaded as follows: for the outgroups, *Homo sapiens* and *Mus musculus* were from Ensembl [24] release 67; and *Bos taurus*, *Canis lupus familiaris*, *Sus scrofa* and *Ovis aries* were from Genbank [25] release 102, 102, 103 and 100 respectively. For the nematodes, *Caenorhabditis elegans* and *Brugia malayi* were from Wormbase [26] WS230; *Trichinella spiralis*, *Dirofilaria immitis*, *Ascaris suum*, *Haemonchus contortus* and *Necator americanus* were from published data [27–31]. *Trichuris muris* was from the Sanger Institute release (ftp://ftp.sanger.ac.uk/pub/pathogens/Trichuris/muris/). *Loa* was from Broad Institute release (http://www.broadinstitute.org). The other 2 nematode species, *Ancylostoma ceylanicum* and *Trichuris suis* were from our in-house sequencing datasets. For the Platyhelminthes, *Schistosoma japonicum* was from Chinese National Human Genome Center at Shanghai (http://lifecenter.sgst.cn/schistosoma/en/schistosomaCnIndexPage.do#Download); *Schistosoma mansoni* was from the Sanger Institute release (ftp://ftp.sanger.ac.uk/pub/pathogens/Schistosoma/mansoni/genome/gene_predictions/GeneDB_Smansoni_Proteins.v4.0g.gz, retrieved on 02/29/2009); *Schistosoma haematobium* was downloaded from SchistoDB (http://SchistoDB.net) [32] on 02/01/2012; and *Clonorchis sinensis* was downloaded from NCBI (NCBI bioproject 72781 [33]). All the kinetoplastids (*Trypanosoma brucei*, *Trypanosoma cruzi*, *Leishmania major* and *Leishmania donovani*) were downloaded from TriTrypDB (http://tritrypdb.org) [34] on 01/07/2014 (release 6.0). *Plasmodium falciparum* was downloaded from NCBI (ftp://ftp.ncbi.nih.gov/genomes/Protozoa/Plasmodium_falciparum/) on 01/07/2014. Isoforms of these downloaded sequences were examined against the coding genes, and only the longest ones were kept when applicable.

### Protein Family Definition and Identification of KDAC Protein Families

Protein families (orthologous groups) were defined utilizing the Markov cluster algorithm [35] of the OrthoMCL package [36, 37] with an inflation factor 1.5 based on the proteomes. Each protein family consists of at least two proteins from one or more species. The gene annotations of KDAC proteins for human in Ensembl, as well as those reported in literature for the pathogens malaria, toxoplasmosis, trypanosomiasis, schistosomiasis and leishmaniasis [10] were used to identify and manually curate the KDAC protein families. The number of proteins in each of these protein families was used to cluster the 26 species, using Manhattan clustering with average linkage using the software package GENE-E (http://www.broadinstitute.org/cancer/software/GENE-E/). A heatmap based on orthologous protein data was generated in MS Excel Version 2010.

### Experimental Compound Screening in Parasitic Species and Mammalian Cell Line

**Fig 1:**
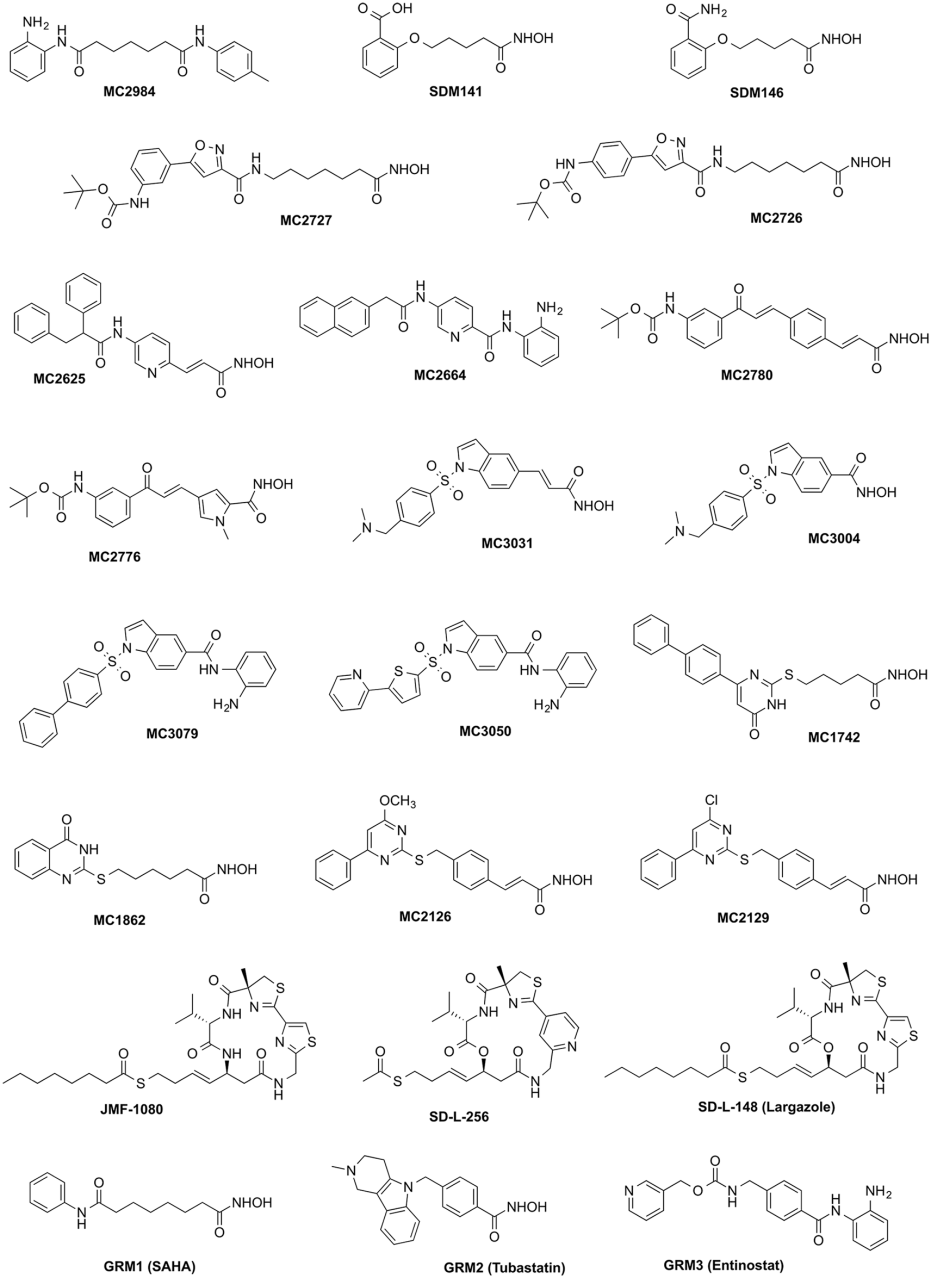
Chemical structures of compounds used in parasite screening assays.

A representative selection of 20 compounds (Fig 1) from a library of several hundred KDAC inhibitors that were synthesized in the Mai laboratory at the Sapienza Universita in Rome was screened. Additionally, largazole and two analogs were supplied by Prof. Robert Williams’ laboratory at Colorado State University. The compounds were selected based on the following criteria: 1). Known KDAC inhibitors which have been well studied and characterized in human studies, usually used as controls, e.g. GRM1 (SAHA), GRM2 (Tubastatin), and GRM3 (Entinostat); 2). Cyclic depsipeptide based, class I-selective KDAC inhibitors and their analogs, e.g. SD-L-148 (Largazole), SD-L-256, JMF-1080; 3). Other hydroxamate- or benzamide-based small molecules which have been shown to be human KDAC inhibitors in purified enzyme-based assays.

Compounds were tested against 3 parasite groups (2 roundworms, 2 protists and malaria, Table 1) and a mammalian cell line (L929 mouse fibroblast; NCTC clone 929 [L cell, L-929, derivative of Strain L] (ATCC CCL-1) was obtained from ATCC). Compound screening against roundworms was conducted using three organisms with very different modes of parasitism: the blood feeding and gut dwelling *H*. *contortus*, and the animal and human tissue-dwelling filarial nematodes *D*. *immitis* and *B*. *malayi*. Experimental procedures were described in previous studies [38, 39]. The kinetoplastids (*T*. *brucei strain S427* and *L*. *donovani strain* MHOM/SD/00/LS) viability assays were conducted with exponentially growing trypomastigotes, oraxenic amastigotes, for each species respectively in 96-well plates using automated liquid-handling equipment. Test compounds in DMSO were added to each well at 2–5 μM for *T*. *brucei* and 5–10 μM for *L*. *donovani* followed by incubation with the parasite for 72 hours at 37°C with 5% CO_2_. Known anti-trypanosomal compounds, i.e. pentamidine and suramin, were included in each plate to serve as positive controls. Parasite viability was determined by addition of resazurin and evaluation of plates using a fluorescent plate reader. Compounds showing ≥75% inhibition in primary assays were selected and titrated to confirm their activity and to generate IC_50_ values. Activity/base protocols were used to calculate IC_50_ values and generate quality control parameters for each plate. Compounds with IC_50_ ≤ 1 μM for *T*. *brucei* and IC_50_s ≤ 5 μM for *L*. *donovani* were tested versus mammalian cells to determine parasite versus host-cell selectivity. A *P*. *falciparum* viability assay [40] was conducted with the 3D7 strain of *P*. *falciparum* known to be sensitive to all antimalarial drugs. Assays were performed in 96-well microtiter plates, and each well contained 100 μl of parasite culture maintained in media supplemented with human red blood cells (0.5% parasitemia, 2.5% hematocrit) in a humidified atmosphere at 37°C, 5% O_2_ and 5% CO_2_. Test compounds in DMSO were added to each well at 2–5 μM. After incubation, 85% of the supernatant was removed and cells were washed with PBS. A DNA-specific dye (SYBR Green or DAPI) was added in the presence of lysis agents, saponin and Triton X-100. Plates were incubated for 15 min and then read in a fluorescent microplate reader. Compounds showing ≥75% inhibition in primary assays were cherry-picked and titrated to confirm activity and generate IC_50_ values. Activity/Base protocols were used to calculate IC_50_ values and generate quality control parameters for each plate. Compounds with IC_50_ ≤ 1 μM were tested against mammalian cells (to determine parasite versus host-cell selectivity) and also against a selection of drug-resistant strains of *P*. *falciparum*.

**Table 1:** Compound screening in host cells and parasites. Only measured activities were reported in the table, in the units of nM. The vertical line in the table separates host and parasites. Abbreviation used: CYT vt: cytotoxicity viability assay; Endoparasites_DR: endoparasites dose response assay; HAT vt: Human African *trypanosoma* viability assay; LEI axe: *Leishmania* axenic amastigote assay; MAL vt: Malaria viability assay. L929: L929 mouse fibroblast; TbbS427: *T*. *brucei* strain S427; Ld1S: *L*. *donovani strain* MHOM/SD/00/LS; PfDd2: *P*. *falciparum* 3D7 strain.

Assay	CYT vt	Endoparasites_DR			HAT vt	LEI axe	MAL vt
Cell line / Species	L929	*B*. *malayi*	*D*. *immitis*	*H*. *contortus*	TbbS427	Ld1S	PfDd2
**Timepoint**		5 day	72 hour	96 hour	72 hour	72 hour	15 minute
MC2984							
SDM141							
SDM146							
MC2727a*	2.35						0.96
MC2726*	1.92						0.189
MC2625*	0.311				1.18	>5	0.022
MC2664							
MC2780*	4.81	2.53	8.14		0.623	0.473	0.056
MC2776*	>10	4.39	>10				
MC3031*	0.555				0.267	>5	
MC3004							
MC3079							
MC3050							
MC1742*	1.51						<0.01
MC1862*	7.12						1.15
MC2126*	1.76				0.441	>5	1.92
MC2129							
JMF-1080							
SD-L-256	0.333	>10	>10	0.9			
SD-L-148	0.101			7.1			
GRM1	0.155				1.81	>5	0.152
GRM2*	6.22				2.7	>5	
GRM3							

* compounds with lower activity in at least one parasite species compared to L929.

### Protein Structural Modeling and Ligand Docking

For those KDAC isotypes in parasitic species targeted by active compounds, homology models were built by using the X-ray structure of the human ortholog as template, using the ROSETTA3.4 macromolecular modeling package [41]. The catalytic zinc ion at the active site was modeled explicitly following the approach of Wang et al to mimic the square-based pyramidal geometry as observed in crystal structures [42][43]. After the initial comparative modeling and loop building, each protein model was relaxed with the following constraints to achieve the desired geometry: the zinc ion was constrained to have the axial position coordinated to the conserved histidine residue (HIS, deprotonated Nε), two equatorial positions coordinated to the conserved aspartic acid residues (ASP, deprotonated hydroxyl oxygen) and the remaining two equatorial positions coordinated to solvent water molecules. 100 models for each target were generated using the constrained relaxation procedure, and the one with the lowest total energy was chosen as the final protein model for subsequent docking studies. For each small ligand to be docked, OMEGA [44] was used to generate a conformer library; OpenEye's AM1-BCC implementation [45] was used to calculate partial charges. The hydroxamate group was deprotonated in the modeling process, as suggested by previous docking and virtual screening reports [46]. The ligands were docked to the models in ROSETTA using the ligand_dock application by specifying a constraint of the hydroxamate group to be coordinated to the zinc, replacing the two water molecules used in modeling the zinc geometry. One hundred poses were generated for each compound at each target, the 5 best-scoring poses were selected for manual inspection, and a representative pose was finally chosen for interpretation.

### Identification of Active Site Variances

For each KDAC protein isotype, a representative X-ray structure from its human ortholog was chosen as the structural template. Any residue with an atom within a distance cutoff (10 Å) to the catalytic zinc ion was defined as an active-site residue. Sequence alignments of other parasite orthologs with the human protein (built by MUSCLE [47] for each KDAC family) were used to identify residues that were different in the parasite, and these residues were identified as variants at the active site.

## Results

### The KDAC Protein Families in the Parasites

Protein families were constructed from all longest isoform sequences in the proteomes of 26 eukaryotic species, which included 20 parasitic species and 6 host species (OrthoMCL clusters with multiple sequences were defined as protein families). The dataset includes 399,592 proteins in total, from which 44,531 protein families were derived. We identified all of the KDAC protein families within the parasitic species, based on the annotations of the human orthologs and the annotations available for a few parasitic species. The annotations of *C*. *elegans* KDAC proteins from WormBase [48] were also used for inference and manual curation.

**Fig 2:**
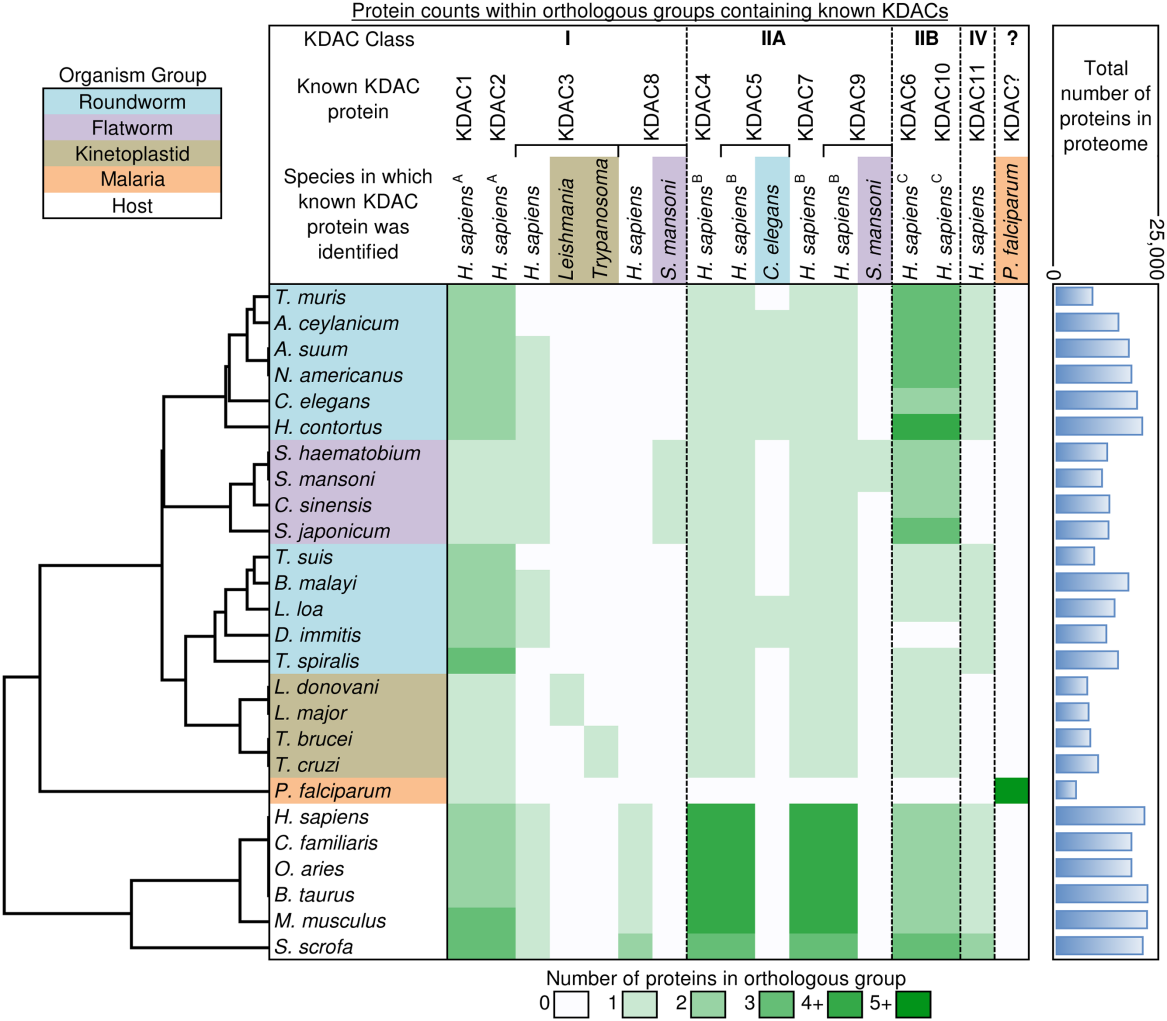
KDAC proteins inferred for the parasitic species within protein families. Those with the same superscripts (A, B, C) are clustered within the same family. Color codes provide the number of total proteins from each species within an orthologous protein family.

As shown in Fig 2, all 11 Zn-dependent KDAC protein isoforms were not present within the parasites. From the human ortholog annotations, all the human KDACs were clustered into 6 separate families. KDAC1 and KDAC2 were clustered into one family (note A), and the class IIA isotypes (4, 5, 7, and 9) and IIB isotypes (6 and 10) were each clustered into their own families (notes B and C). Isotype KDAC3 from most species was clustered into one family (as was KDAC11), while KDAC8s from all the hosts were clustered in one family.

In the class IIA family, almost all of the parasites had only one member in the family containing human orthologs, except *P*. *falciparum*, which had no member present. Based on the *C*. *elegans* annotation, there should be orthologs of KDAC4 for the roundworms. Some roundworms (*A*. *ceylanicum*, *A*. *suum*, *N*. *americanus*, *H*. *contortus*, *L*. *loa* and *D*. *immitis*) also have the KDAC5 ortholog, which clustered with the *C*. *elegans* KDAC5 into a new protein family. Primary sequence similarity results suggested that the kinetoplastids share higher homology to the human KDAC5 protein, so these were annotated as KDAC5. Two flatworm species (*S*. *mansoni* and *S*. *haematobium*) also had a KDAC9 ortholog, both of which were clustered into a separate protein family. From the current analysis, no KDAC7 ortholog was present in any parasitic species examined.

Although there are only two isotypes in the class IIB family, two human protein members (KDAC6/10) showed considerable expansion/deletion among the parasites. The kinetoplastids only contained one member, while many of the roundworms and one of flatworms (*S*. *japonicum*) have three or more members. Some of the roundworms had only one ortholog in this family, with one of them (*D*. *immitis*) having no member present.

For the other isotypes, a single KDAC3 homolog was uniquely shared among *Leishmania* species (and another among *Trypanosoma* species), with all of the hosts and flatworms and most of the roundworms sharing orthology to the human KDAC3 protein, suggesting divergence of this protein among the kinetoplastids. Orthologs to the human KDAC8 were only found among host species, but a separate *S*. *mansoni* KDAC8 orthologous group was found to be conserved in (and uniquely found in) flatworms. Finally, KDAC11 was conserved only among host and roundworm species.

Based on the number of protein members within the protein families, the different species were clustered according to their phylogenetic distances to each other (Fig 2). Kinetoplastids, flatworms and hosts were all clustered with their own species groups, but the roundworms clustered into two groups, largely due to the expansion in the number of KDAC6 and KDAC10 orthologs among the roundworms in the top cluster. *P*. *falciparum* clustered separately from all of the other species, as it contained only one protein (124507060) with detected orthology to any KDACs identified in other species. This protein was orthologous to KDAC1/2 family members, previously characterized as a KDAC1 ortholog [49]. Two of the three other KDAC homologs previously identified in *P*. *falciparum* [10] are orthologous to each other, but share no orthology to any other species examined here. There are far fewer proteins in the *P*. *falciparum* proteome compared to the other species (just 5,337 proteins compared to more than 8,000 in every other species), but a smaller number of proteins did not necessarily limit the representation of KDAC orthologs, as seen in the roundworm species in Fig 2.

### In Vitro Screening of Compounds against Various Parasite Species

For compound screening, we adopted cell-based approaches, since these compounds have been previously reported to have activities in isolated enzyme assays on human KDAC proteins. A total of 13 compounds out of the 23 screened showed efficacy in at least one parasite, with all of them also showing some kind of activity on the mammalian cell lines (Table 1). Most of the active compounds showed extremely high (nM to sub-nM IC_50_) potency in the inhibition of *P*. *falciparum* growth. This was consistent with reports for the hydroxamate-based KDAC inhibitors acting on the malaria pathogen [12]. Approximately 10 compounds had IC_50_/E_50_ lower (ratio < 0.5) in at least one parasite species compared to the host-cell line. One of the compounds (**MC2776**), a pyrrole-based hydroxyamate derivative, shows considerable potency (EC_50_ = 4.39 μM) on the nematode *B*. *malayi*, without detectable activity in the host-cell line (> 10 μM), making it a candidate for further optimization and in-depth study. The activities of **MC2776** and **MC2780** observed in our work were in accord with those reported recently (**8b** and **10c** therein) [50], with higher than 10 μM IC_50_ on human cancer cell lines for **MC2776 (8b)** and more potent IC_50_ for **MC2780 (10c)**. None of the benzamide analogs showed activities on these cell lines (> 20 μM), although *in vitro* activities of these compounds was reported on human KDACs [51]. This suggested a possible role for the hydroxyamate/benzamide group on cell permeability/transport.

### Homology Models and Ligand Binding to the KDAC Proteins

**Fig 3:**
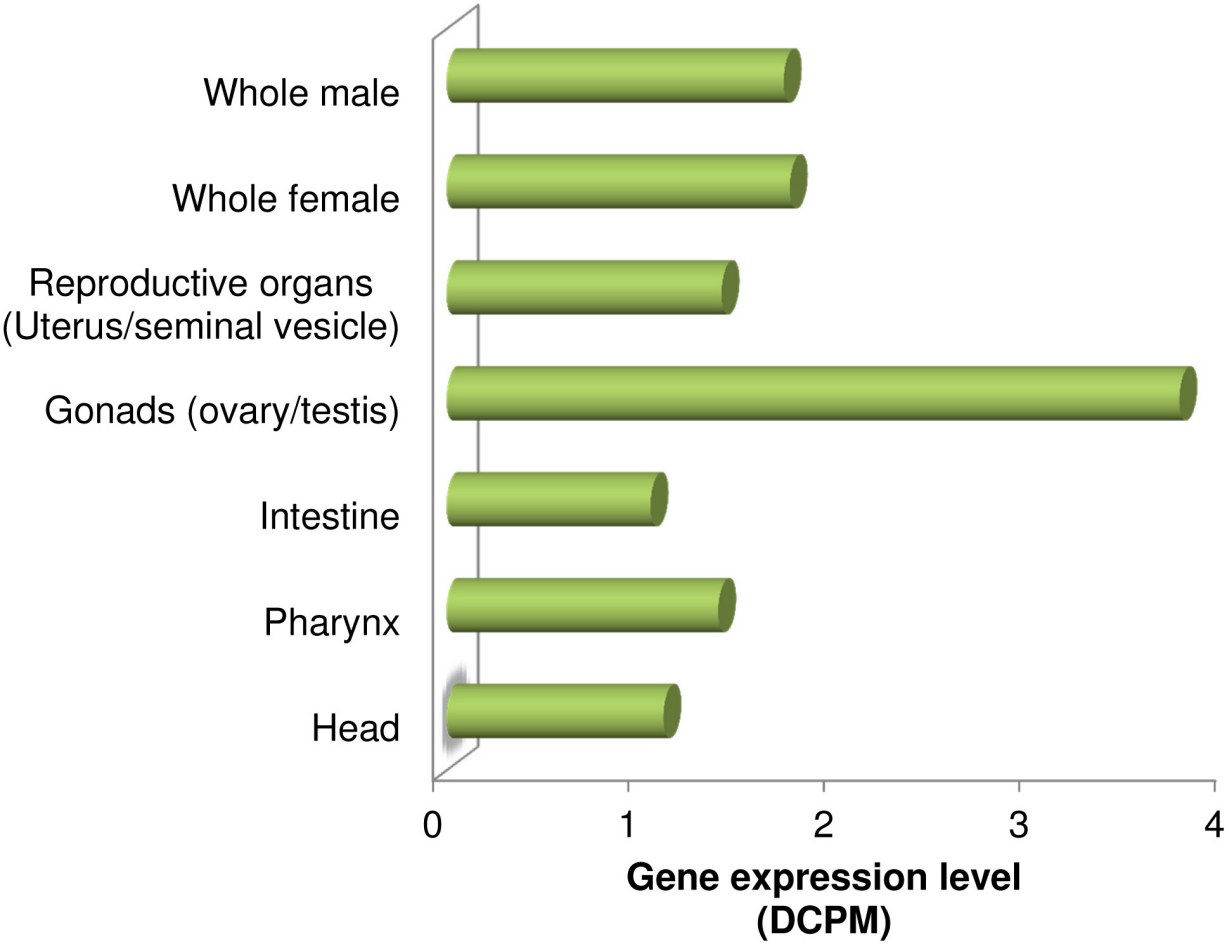
Expression level of *A*. *suum* KDAC1 gene (GS_10652) in different tissues. Gene expression values are in depth of coverage per million reads (DCPMs). The expression values are averaged across male and female samples.

The active compounds from *in vitro* screening were used to study ligand binding to the KDAC proteins, using computational methods. Two of the representative compounds, **MC2776** and **MC2780**, were docked to the KDAC1 isotypes of the host (human) and each of the parasitic species (*B*. *malayi*, *L*. *donovani* and *P*. *falciparum*). The KDAC1 isotype was chosen because it is ubiquitously expressed in all tissues within all the organisms studied, as reported recently in a tissue-specific expression profiling for 10 *A*. *suum* tissues (Fig 3; [52]). In addition, the recently reported X-ray structure of the human protein (pdb code: 4BKX [53]) facilitates structural modeling of the parasitic orthologs with side-by-side comparison of ligand binding.

Homology models were built for the KDAC1 orthologs from three parasitic species (*B*. *malayi*, *L*. *donovani* and *P*. *falciparum*) respectively using the human crystal structure as template. The sequence identity and similarity between each target and the template are high, especially for the nematode *B*. *malayi* (S1 Table), suggesting the models should have adequate resolution for the subsequent docking study. The RMSD (root-mean-square-deviation) values for each model after each step in the modeling process remain stable at below 2Å, indicating that the models show high similarities to the human structure, and that there are only subtle differences in the loop regions and side chain conformations which may lead to differences in binding modes.

**Fig 4:**
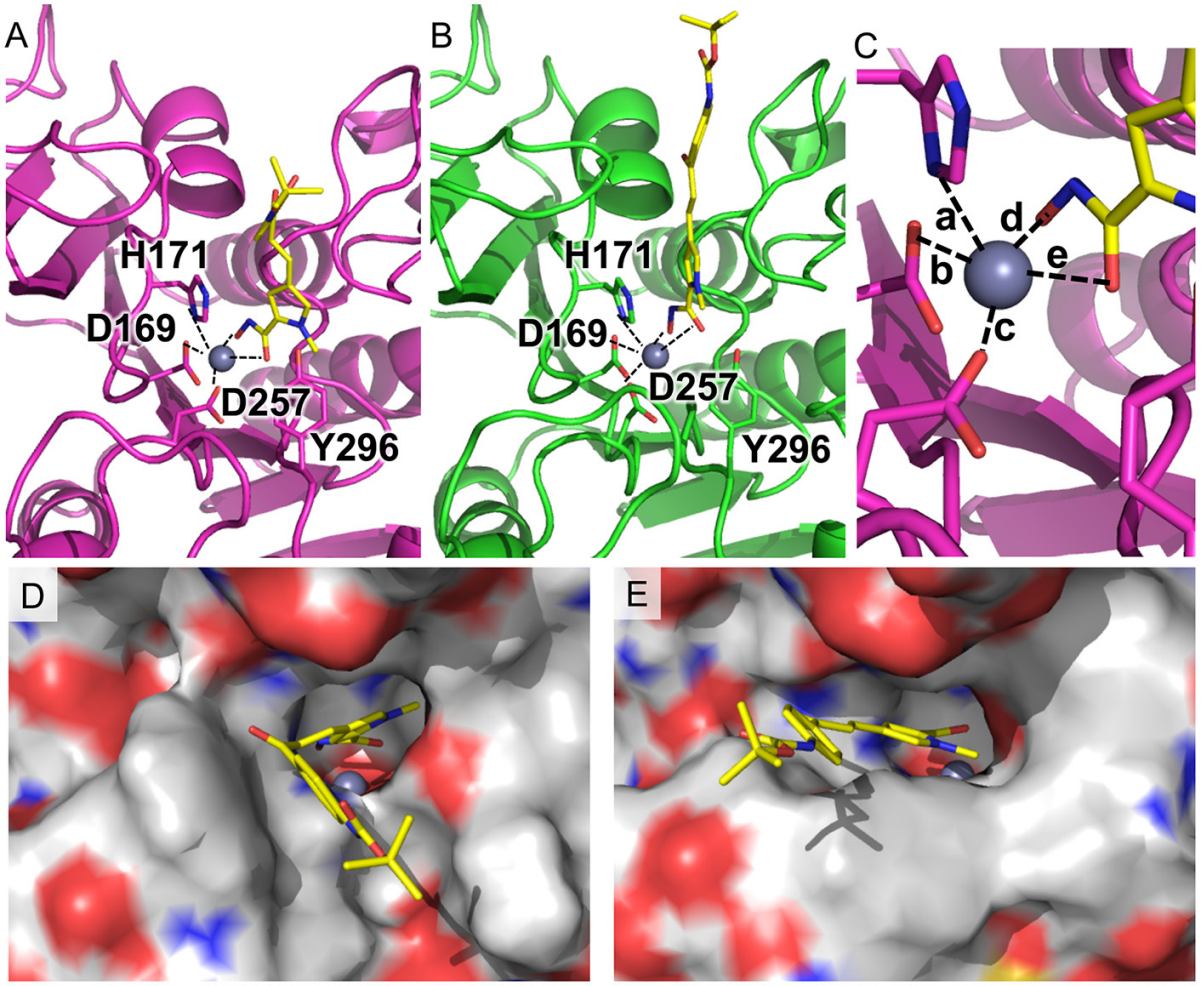
Compound MC2776 docked to the KDAC1 protein. **(A) in the *B*. *malayi* protein and (B) in the *H*. *sapiens* protein.** MC2776 is shown as yellow stick model along with important residues for ligand binding. (C) shows a close-up view of the zinc-centered square based pyramid, Distances for these are shown in Table 2. (D) and (E) show the rendered surface models of the cartoon representations from (A) and (B).

**Fig 5:**
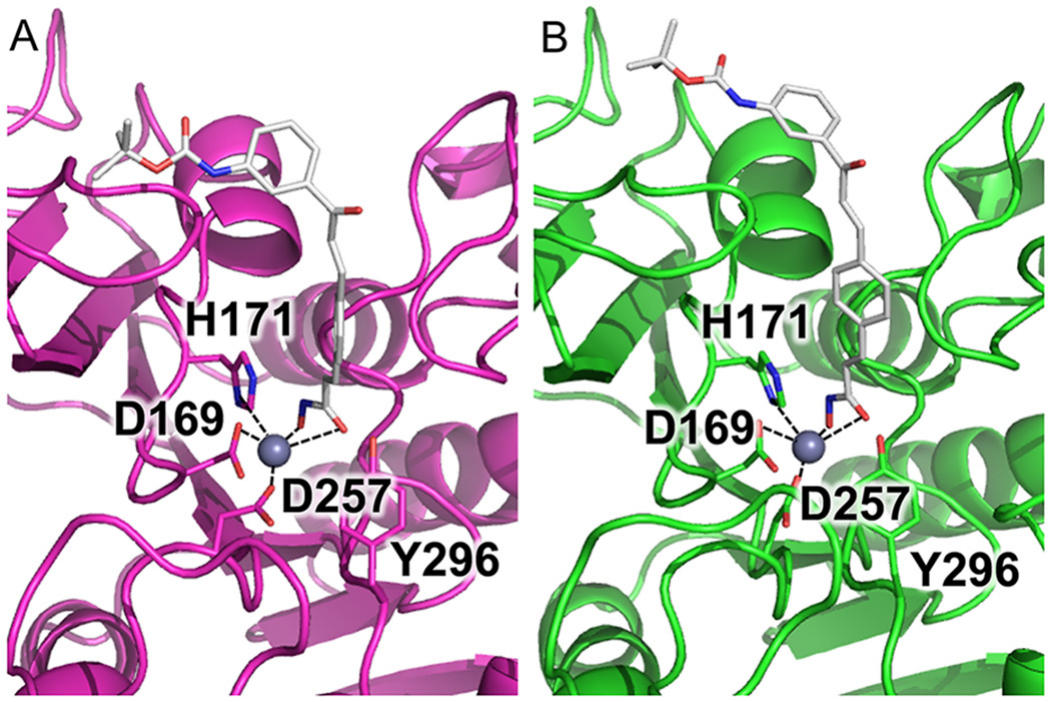
Compound MC2780 docked to the KDAC1 protein. A) in the *B*. *malayi* protein and B) in the *H*. *sapiens* protein. MC2780 is shown as grey stick model along with important residues for ligand binding.

To validate our docking procedure, a benchmark docking study was also performed for the crystal structure, using the bound ligand (acetate ion). The experimental pose was successfully obtained for acetate (RMSD between lowest energy ligand conformation and crystal structure: 0.74 Å). This validates the potential utility of the docking procedure. The subsequent docking of the two ligands from the screening suggests that both ligands could bind relatively well with the orthologs, but shows some differences at the different ortholog binding sites, especially for the roundworm-selective ligand **MC2776** (Fig 4). The models showed that when viewed from above, the ligand the pyrrole ring was almost perfectly in the plane of the hydroxymate in human KDAC1 with the hydroxymate group chelated with catalytic zinc. However, in the *B*. *malayi* ortholog, the pyrrole ring rotated counter-clockwise in order to accommodate the tyrosine residue (Y296) at the opening at the binding channel (Fig 4a). The tyrosine residue is conserved across all the KDAC isotypes among almost all organisms, and has been implicated to play a critical role in the selective ligand binding to KDAC8 of *S*. *mansoni* [16]. The different orientation of the Y296 in *B*. *malayi* could be attributed to a nearby point mutation (C254N). The small hydrophobic residue in other species is tightly packed beneath the binding pocket; while in *B*. *malayi*, the bulkier, more hydrophilic asparagine led to a propagation of rearrangements of the two strands nearby, resulting in a misaligned tyrosine residue at the protein surface. In contrast, because of the lack of the pyrrole ring, **MC2780** showed very similar binding modes in the KDAC1 proteins of human and *B*. *malayi* (Fig 5). The tert-butylcarbamate group at position 3 of the terminal phenyl ring extended toward the outer portion of the binding gorge, contacting one of the loops lining the rim of the catalytic tunnel (residues G677–G686), while in the protist proteins, the same group tilted away to the other side of the channel (S1 Fig). The different binding modes of **MC2776** and **MC2780** at KDAC1 may partially explain the different affinities among different organisms. Although quantitative binding energies cannot be obtained from simple docking simulations, the distances of the catalytic zinc atom to its coordinating atoms from the protein and ligand were measured in all the models for comparison (Table 2). In general, the ortholog protein with higher binding affinity with the ligand shows shorter distance (both mean and standard deviation) to the zinc atom for most of the coordinating atoms, to maintain the optimal square-based pyramidal geometry. Docking results showed that both **MC2776** and **MC2780** could bind the KDAC1 of the protists (*L*. *donovani* and *P*. *falciparum*); However, **MC2776** did not show any efficacy in any of the protists, while **MC2780** demonstrated pan-parasite potential. This may be due to its inability to reach its target under assay conditions due to metabolism, transport, or other issues [54].

**Table 2:** Distances of the catalytic zinc atom to the binding residue and ligand chelating atoms in the models of MC2776 and MC2780 docked to KDAC1 protein of different species. The distances shown are in the unit of Angstroms. The chemical formulae for both ligands are depicted in Fig 1. O1 and O2 are from hydroxamate group of the ligands. Other atoms are from the protein, as shown in Figs 4 and 5. The coordinates of the models as pdb file are available in S1 Supporting Materials.

Compound	MC2776				MC2780			
**Species**	*H*. *sapiens*	*B*. *malayi*	*L*. *donovani*	*P*. *falciparum*	*H*. *sapiens*	*B*. *malayi*	*L*. *donovani*	*P*. *falciparum*
ZN-ND1 (His171) (a)	3.18 ± 0.47	2.80 ± 0.04	2.51 ± 0.18	2.64 ± 0.12	2.76 ± 0.09	2.75 ± 0.09	2.30 ± 0.05	2.66 ± 0.15
ZN-O (Asp169) (b)	2.26 ± 0.13	2.22 ± 0.05	2.43 ± 0.03	2.39 ± 0.07	2.12 ± 0.04	2.17 ± 0.03	2.41 ± 0.02	2.46 ± 0.11
ZN-O (Asp257) (c)	2.68 ± 0.31	2.18 ± 0.08	2.17 ± 0.08	2.18 ± 0.06	2.44 ± 0.03	2.16 ± 0.06	2.09 ± 0.09	2.20 ± 0.04
ZN-O1 (Ligand) (d)	2.24 ± 0.29	2.08 ± 0.02	2.06 ± 0.06	2.09 ± 0.05	2.04 ± 0.01	2.11 ± 0.04	2.05 ± 0.04	2.13 ± 0.08
ZN-O2 (Ligand) (e)	2.59 ± 0.41	2.26 ± 0.05	2.83 ± 0.41	2.48 ± 0.26	2.62 ± 0.15	3.09 ± 0.08	3.10 ± 0.21	2.21 ± 0.11
EC50(IC50) (mM)	>10	4.39	-	-	4.81	2.53	0.47	0.06

### Active-Site Variance of the KDAC Proteins for Selective Ligand Design

To facilitate selective ligand design, we systematically examined all of orthologous proteins within the parasite species and identified the variant residues near the active site for comparison with human protein structures. X-ray crystal structures of the catalytic domain have been reported for 6 of the 11 zinc-containing KDAC isotypes in human, i.e. KDAC1, 2, 3, 8 (class I) and 4, 7 (class II). Among them, KDAC7 lacks a clear ortholog in any of the parasite species, so active-site variants were reported for KDAC1, 2, 3, and 8 together with the only class II isotype KDAC4 (Table 3), showing that active-site residues were well conserved in most of parasite orthologs (especially for KDAC1). The kinetoplastid orthologs have slightly more variance, with 8 out of the 50 residues being different from the human protein. All the other orthologs of KDAC1 in the parasite proteins had 5 or fewer residues different from the human protein, with the exception of *B*. *malayi*, which had 6 different residues. Interestingly, the one uniquely different residue (C254N) is the also a contributing factor of the different binding mode of MC2776, as suggested by docking. This demonstrates that variation at active sites could play an important role in the pursuit of selective ligands. It is not surprising to see a higher variances for KDAC3 in the kinetoplastids, since each of the two genera has been grouped into its own orthologous protein cluster (*Leishmania* and *Trypanosoma*), suggesting their divergent distance from the human ortholog. KDAC8s of trematodes also clustered into a separate cluster than the mammalian orthologs, but still showed quite high conservation at the active sites, with only 8 out of the 48 residues different. In the class IIA family cluster, only one member was found for the parasites except for *P*. *falciparum*. Annotated KDAC4 proteins (roundworms and flatworms) were compared with the human KDAC4 structure and were well-conserved except for *A*. *ceylanicum*, with 22 different residues out of a total of 49. A closer examination revealed that this was due to the fragmented sequence within that region, since at positions 21, there are gaps instead of amino acid residues. This is likely an artifact resulted from the draft nature of the genome (proteome) data.

**Table 3:** Sequence variations of KDAC proteins of parasitic species in comparison with the host (*H*. *sapiens*) orthologs at the active sites. Active-site residues were defined as any residue with a distance less than 10 Å to the catalytic zinc in the crystal structure. Abbreviations were used for the names of all the species following the rule of “the first letter of genus + first three letter of species”.

Class		I				IIA
Target Protein		KDAC1	KDAC2	KDAC3	KDAC8	KDAC4
Human gene (*H*. *sapiens*; ENSG00000XXXXXX)		116478	196591	171720	147099	68024
PDB code		4BKX	4LXZ	4A69	1T67	4CBY
Total defined residues		50	49	50	48	49
Nematode	Acey	1	-	3	-	22
	Asuu	1	0	3	-	5
	Bmal	6	0	2	-	13
	Cele	5	3	3	-	5
	Dimm	2	0	4	-	6
	Hcon	1	1	3	-	5
	Lloa	2	0	4	-	6
	Name	1	12	3	-	5
	Tmur	4	-	4	-	14
	Tspi	3	-	4	-	9
	Tsui	3	-	3	-	13
Kinetoplastid	Tcru	8	-	21	-	-
	Tbru	8	-	26	-	-
	Lmaj	8	-	14	-	-
	Ldon	8	-	14	-	-
Malaria	Pfal	3	-	-	-	-
Trematode	Sman	4	-	3	8	14
	Sjap	1	-	3	8	7
	Shem	1	-	3	8	9
	Csin	4	-	6	8	7

## Discussion

We presented the first systematic examinations of all zinc-dependent KDAC proteins in representative parasites from nematode, trematode, kinetoplastid and malaria pathogens, and showed that some human KDAC isotypes lack clear orthologs in the parasites, with the only conserved isotype across all the species studied being KDAC1. KDAC1 enzymes are primarily localized in the nucleus and are expressed in all tissues almost ubiquitously. As a classical KDAC protein, it has been studied extensively both in human and in the parasites such as *P*. *falciparum* as a novel drug target [6, 12]. KDAC1 shares high sequence similarities among all the species studied. It has been reported that KDAC1 of *P*. *falciparum* shares over 55% sequence identity to yeast, human, chicken, and frog KDAC orthologs [55]. This also consistent for the nematode/trematode KDAC1 in comparison with host orthologs, but the homology was lower in the kinetoplastids, which had ~40% sequence identity with the remaining species (S2 Fig). It was also reflected in active-site residues, with most of the variants coming from the kinetoplastid orthologs. As shown in S2 Fig, out of the 12 active-site variants from all the parasite proteins, 3 are specifically present in the kinetoplastids, indicating a higher level of divergence. It has been found that the KDACs of kinetoplastids branched very early from the eukaryotic lineages, especially for the class I isotopes [56]. This suggests that it would be relatively easier to design selective compounds against the kinetoplastid KDAC1. Achieving species selectivity within the other parasite species would still be difficult, due to the higher level of conservations of protein sequence especially at the active site. In this regard, the molecular ligand binding modeling of *B*. *malayi* KDAC1 offered potential insight on improving parasitic selectivity. In fact, a BLAST search of the protein sequence against other nematode proteomes suggests that the C254N mutation was present within KDAC1 of other *Brugia* species (*B*. *timori* and *B*. *pahangi*), as well as the *Onchocerca* species (*O*. *ochengi*, *O*. *flexuosa*, and *O*. *volvulus*).

The other members in the class I isotype were tentatively labeled as KDAC3 for the kinetoplastids, although they were each clustered into their own families (*Leishmania/Trypanosoma*). They showed even greater divergence in comparison with other species, raising the possibility that they were completely new isotypes instead of KDAC3 orthologs. Nevertheless, given their divergence from any other KDACs, it might be worthwhile to pursue their roles as selective drug targets. Much work has been done on the trematode KDAC8 (specifically in *S*. *mansoni*) as a novel drug target for the control of schistosomaisis, since it is the most highly expressed class I KDAC isotype in this organism [16]. Our results indicate that KDAC8 is in a separate family of KDACs within the flatworms, with some divergence from host orthologs. Structural characterization of *S*. *mansoni* KDAC8 confirmed our findings and supported the observation that selective ligands can be designed to explore subtle conformational differences at the active site [16].

Interestingly, among the class II families, IIA and IIB proteins showed different gene expansion/deletion patterns within the parasite species compared to hosts. Four members have been identified for the class IIA proteins in the hosts (4, 5, 7, and 9), while only 1 or 2 members were present in the parasites. In contrast, in the class IIB families, some parasites (especially the roundworm species) showed considerable expansions. This has been observed before in the non-parasitic nematode *C*. *elegans* [57] and was confirmed in many of the parasitic roundworms analyzed in this study. However, some of roundworms (like all the protists) also showed a loss of genes, none or just one protein among the two class IIB protein families (6, 10). The highly variable gene expansion/loss pattern implied different functional roles for the class IIB isotypes in nematodes, which is a topic worth further exploration.

The much less-studied KDAC11, as the sole member of the class IV isotype, was only present in hosts and nematodes. KDAC11 has been found to be differentially expressed among different tissues and was suggested to be a novel drug target in human carcinomas [58]. Further characterization of its roles in nematodes could decipher its specificities and reveal any potential for targeted therapeutics. It was surprising that two of the class II KDACs in *P*. *falciparum* were clustered into a completely new protein family, with none of the known KDAC proteins from other species present. This protein family contained the most members (1271 proteins) among all the families generated, including 1244 *P*. *falciparum* proteins and 27 proteins from the other 12 species. Besides the two KDACs, the 1244 *P*. *falciparum* proteins within this protein family were assigned annotations ranging from protein enzymes (kinase, polymerase, protease, transferase, and etc.) to transporters, ion channels and many others, with over half of them annotated as unknown functions. This indicates that the protein family was a promiscuous assembly with no consensus functions identified, and also showed that the two *P*. *falciparum* KDAC proteins have the largest evolutionary distances from other species (consistent with previous observations [10, 12]).

In this work, we report some preliminary results of compound screening against a panel of parasitic species, and explored the mechanisms of their activities at the molecular level for certain KDAC isotypes. Docking calculations were performed on the homology models for all the parasitic protein orthologs. Caution should be exercised when interpreting the observations made for protein-ligand interactions, since homology models have limited representation of different loop conformations between the target structure and the template. In our case, the sequence identity between template and target was relatively high (ranging from 45% for *L*. *donovani* and 62% for *P*. *falciparum*, to 72% for *B*. *malayi* for the full length, and over 80% for all the species at the active site as shown in Table 3 and S1 Table and S2 Fig), so the docking result in homology models should be reliable at this level [59]. However, it is still premature to draw conclusions based on the docking results on a single isotype, since the "inactivity" of compounds in a full-organism assay may be attributed to a number of possible explanations including: (i) the compound not penetrating the cell (inability to reach the target), (ii) the compound being metabolized in the organism, active excretion from the cell, or (iii) the target of the compound not being essential. In addition, although KDAC1 is the most important target across all the organisms (since it is ubiquitous), the compounds’ molecular interactions on other KDAC isotypes or even other proteins were not examined in detail. The work reported is "preliminary" evidence of KDACs as drug targets, nevertheless it should inspire both biochemical experimental and computational studies, for more detailed characterization of KDAC targets within the parasite species and help generate new and improved selective compounds to target parasitic disease.

### Conclusions

A systematic study of all KDAC proteins within parasitic species from protists to nematodes as novel drug targets is reported. Although much work is still required to elucidate their functions and essential nature of these KDACs in parasites, preliminary compound screening suggests that they are inhibited by known host KDAC ligands; such compounds also showed different degrees of selectivity within the parasitic panel. Molecular modeling in combination with genomic profiling offered insight in the mechanism of selectivity and suggested future directions for selectively targeting parasitic lysine deactylases.

## Supporting Information

S1 Fig Compound MC2780 docked to the KDAC1 protein *of P*. *falciparum*. MC2780 is shown as grey stick model along with important residues for ligand binding as Fig 5.
(TIFF)

S2 Fig Sequence alignments of all the KDAC1 proteins of parasitic species within the family, along with the sequence of *H*. *sapiens*. Active-site residues (within 10 Å distance to catalytic zinc atom) in the crystal structure (4BKX, chain B) are marked as “+” underneath. Any variant residues within the parasitic species are marked as “*” at the bottom.
(TIF)

S1 Table Sequence identity and similarity between each parasite species target and the template (human) structure, as well as the RMSD values in each step of the modeling process. (XLSX)

S1 Supporting Materials A compressed file containing protein data bank-format file for the top 5 scoring poses of docked ligands to the KDAC1 structures for the 4 species (*H*. *sapiens*, *B*. *malayi*, *L*. *donovani* and *P*. *falciparum*) in pdb format. (ZIP)
