# Supplementary figures and images for: Targeting Lysine Deacetylases (KDACs) in Parasites
Source: PLoS Negl Trop Dis. 2015 Sep 24;9(9):e0004026. doi: 10.1371/journal.pntd.0004026 (PMC4581690; doi:10.1371/journal.pntd.0004026)

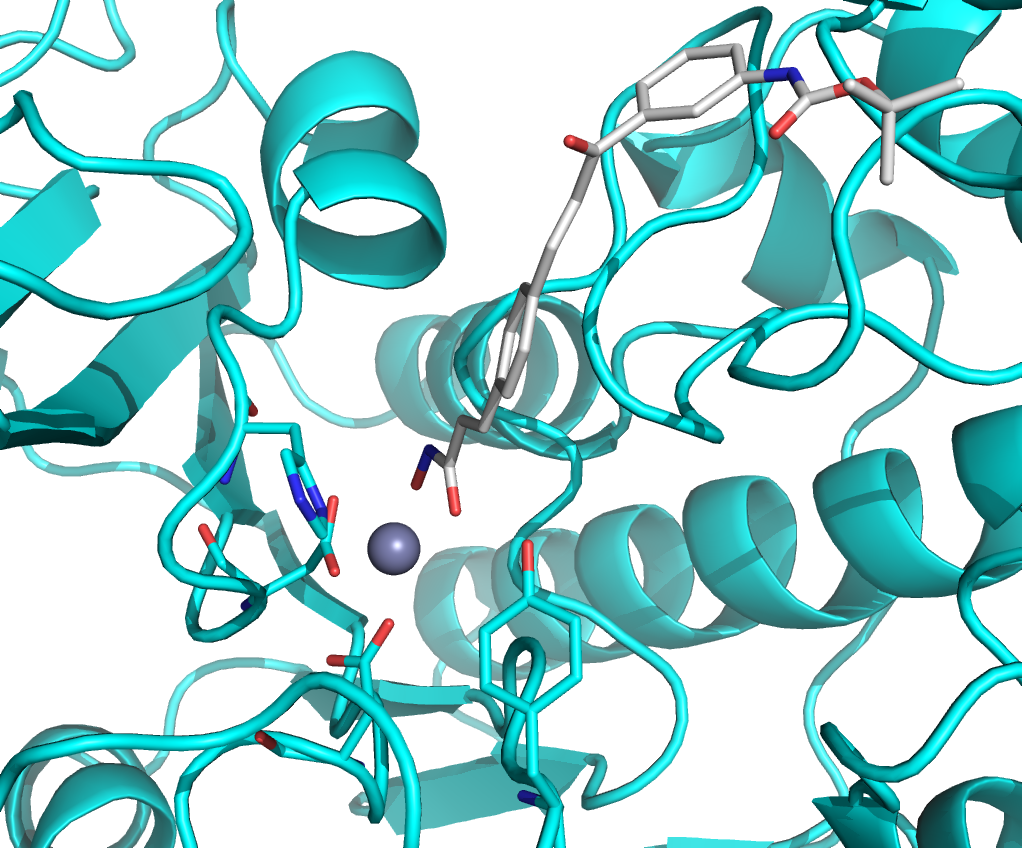

Supplement: S1 Fig — MC2780 is shown as grey stick model along with important residues for ligand binding as Fig 5. (TIFF) [file pntd.0004026.s001.tiff]

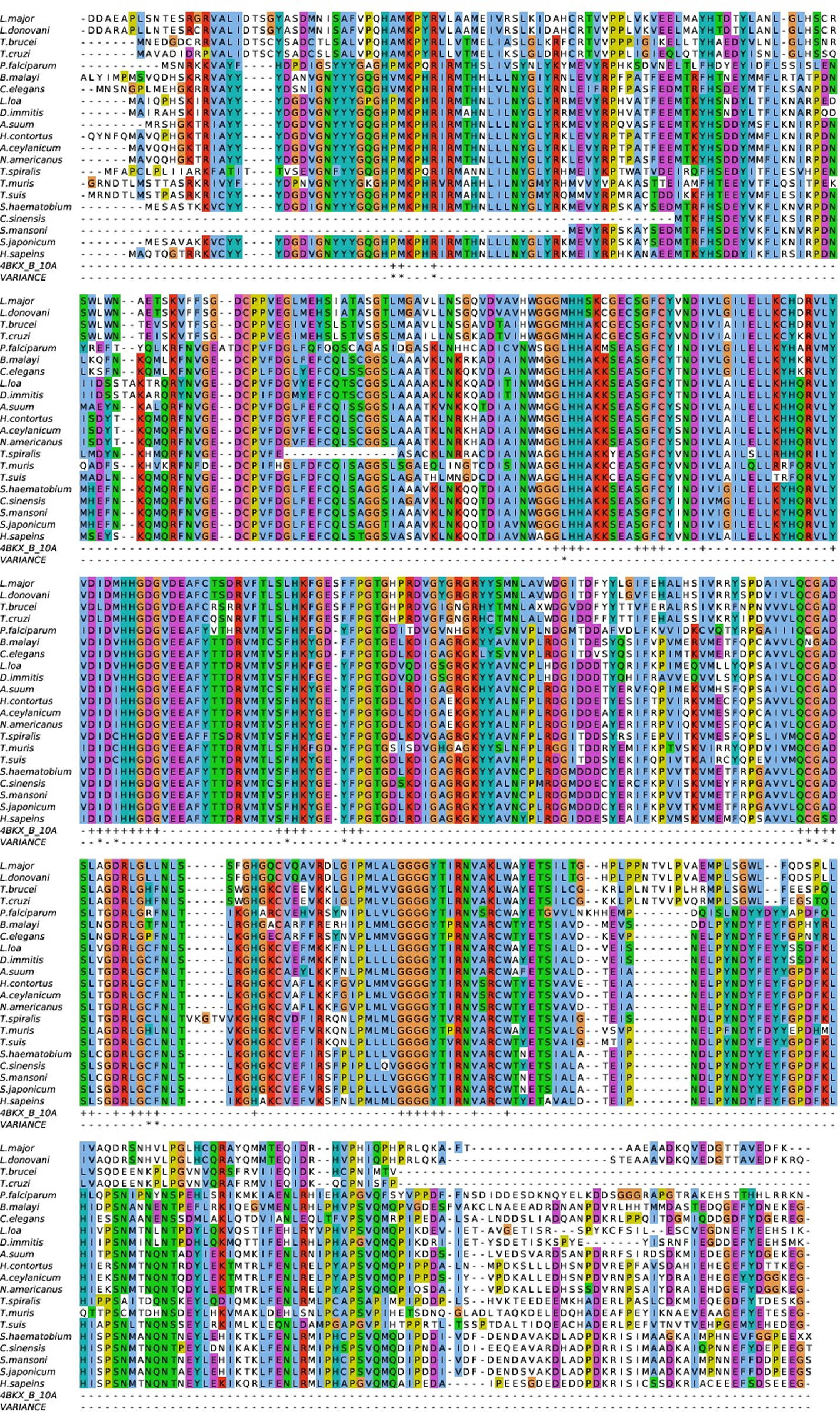

Supplement: S2 Fig — Active-site residues (within 10 Å distance to catalytic zinc atom) in the crystal structure (4BKX, chain B) are marked as “+” underneath. Any variant residues within the parasitic species are marked as “*” at the bottom. (TIF) [file pntd.0004026.s002.tif]
